# Evaluation of results after distal metatarsal osteotomy by minimal invasive surgery for the treatment of metatarsalgia: patient and anatomical pieces study

**DOI:** 10.1186/s13018-019-1159-0

**Published:** 2019-05-08

**Authors:** Miguel Lopez-Vigil, Santos Suarez-Garnacho, Vanesa Martín, Carmen Naranjo-Ruiz, Carmen Rodriguez

**Affiliations:** 1 Department of Morphology and Cell Biology, Faculty of Medicine, University of Oviedo, Julian Claveria, 33006 Oviedo, Spain; 2 San Vicente Martir Catholic University of Valencia, Valencia, Spain

**Keywords:** Second metatarsal, Osteotomy, Minimal invasive surgery (MIS), Shortening, AOFAS

## Abstract

**Background:**

Metatarsalgia of the lesser toes is a common cause of consultation in the podiatric clinic. However, there continues to be a controversy with respect to which is the best surgical technique, and there is few information in the literature regarding objectively comparable results in percutaneous surgery.

**Methods:**

The second metatarsal bones of 30 feet belonging to patients who had attended the podiatric clinic were studied before and after distal metatarsal pecutaneous osteotomy. The degree of shortening of the second metatarsal (RX) and the degree of functional recovery and perception of the well-being of the patient (AOFAS) were evaluated retrospectively.

The same bones of 10 cadaveric feet were also studied. The surgical procedure was identical to that used on patients, and electronic callipers were employed to take measurements of the second metatarsal. The integrity of the plantar plate was checked visually.

**Results:**

The mean shortening of the second metatarsal bone, as determined by the radiological study, was 2.76 mm. After an average follow-up period of 1.5 years, the final mean score on the AOFAS scale was 95.26 points. In none of the cases was the mobility of the metatarsophalangeal (MTP) joint affected. The mean shortening in the cadaveric feet was 2.10 mm, and in all cases, the plantar plate and flexor apparatus were perfectly preserved.

**Conclusions:**

Percutaneous osteotomy achieved, in our study, a lower degree of shortening than Weil’s surgery, according to the data published in the literature. However, it shows good clinical results without causing problems of consolidation or rigidity in the MTP joint. Neither, with the caution that should be taken due to the use of experimental cadaver models, damage of the flexor apparatus of the foot is observed. These results suggest that this could be a safe and effective surgical procedure to be considered for metatarsalgias of the lesser rays.

## Background

Among problems affecting the forefoot, metatarsalgia of the lesser toes is a common cause of consultation in the podiatric clinic. It may be the outcome of numerous disorders but is most frequently associated with mechanical alterations of the forefoot, such as claw toe and insufficiency of the great toe or hallux valgus [1–5]. It is characterised by plantar pain associated with persistent hyperkeratosis under the head of one or various metatarsals of the foot. When it does not improve with conservative measures such as local relief by means of orthopaedic inserts for metatarsal supports, modification of footwear, the use of flexible orthoses to reduce deformities of the toes, anti-inflammatory treatment or multidisciplinary treatment together with physiotherapists, it may require surgical treatment. However, there continues to be controversy with respect to which is the best surgical technique.

Historically, a variety of such techniques have been described for the treatment of metatarsalgias [6–9]. Distal osteotomies were proposed by Borggreve in 1949, then by Davidson [10] in 1969, and by Weil in 1992. The latter was popularised in Europe by Barouk [11] and is nowadays one of the most frequently employed procedures. It is an osteotomy parallel to the load bearing surface of the head of the metatarsal and is fixed with screws, which allows axial decompression.

Percutaneous surgery, also known as minimal Invasive surgery (MIS), is a procedure that allows the surgeon to operate through minimal incisions, without direct exposure of the surgical field, which leads to minimal trauma to the adjacent tissues and requires radiological visualization during the operation, to allow the surgeon to exert precise control over the surgical manoeuvres [12]. Percutaneous surgery cannot be considered a minor procedure simply because it is carried out through small incisions, since via these small incisions, major and even aggressive surgical techniques can be performed [13]. Therefore, it is of fundamental importance that these operations are conducted by expert surgeons [14] and that both the pre-operative preparation of the patient and the measures for the maintenance of sterility are executed with extreme care. Although the history and the results obtained by metatarsal conventional surgery are proven and widely accepted [15–19], the use of MIS procedures to perform a distal osteotomy of the lesser metatarsal maintains the anatomical structures that stabilise the bone ends, since they do not require the dissection of the soft tissues, neither do they demand the separation of the periosteum in the area of the osteotomy. The preservation of these structures allows a good level of vascularisation. Tension of unharmed soft tissues, together with the direction of the osteotomy (from distal-dorsal to proximal-plantar) makes osteosynthesis not necessary. Furthermore, this surgical technique has advantages that can be considered fundamental and which are in line with new surgical trends in other anatomical areas. Among these positive factors, it is worth highlighting the following: this is an outpatient procedure; it uses local anaesthetic accompanied by sedation under the control of the anaesthetist; osteosynthesis material is not required, in contrast with other procedures described here [20]; and finally, there is less postoperative pain and a decreased risk of infection [14], so it can be used in all types of patients, including the diabetic patients with all the problems that they can bring associated [21]. The aim of the osteotomy is the shortening of the metatarsal and the raising of its head to off-load the forefoot reducing the pressure that is causing the pain [22]. Although there are studies that provide information on the shortening achieved in Weil surgery, there are very few data on this shortening and even few clinical studies, after the osteotomy through percutaneous surgery.

Among the soft tissues surrounding the metatarsal head, it is worth mentioning the plantar plate. This is a plantar fibrocartilaginous thickening of the articular capsule, which keeps the toe in the correct position with respect to the joint, avoiding hyperextension and ensuring that the toe does not exceed its normal range of movement [23]. The plate and the collateral ligaments form a sheath of soft tissue connected to the sides of the head of the metatarsal. Besides its role on the stability after surgery, tension of the soft tissues surrounding the metatarsal head determines its final optimal disposition, so it is relevant that these tissues are not damaged during the operation.

The objectives of this study were (1) to analyse the results of distal osteotomy of the second metatarsal bones carried out by a MIS procedure, specifically analysing the metatarsal shortening and the patient well-being and (2) to ensure, as much as it can be done using a cadaveric model, that the plantar plate and soft tissues around the metatarsal head remain unharmed after the operation, as long as this is correctly performed.

## Material and methods

A retrospective study was performed analysing the surgical results obtained after MIS procedure for the treatment of patients older than 18 years who attended the podiatric clinic with metatarsalgia of the central metatarsal bones which was susceptible to surgical treatment (*n* = 30). Minors and patients who had previously undergone surgery to the metatarsals in question were excluded from the study. Informed consent had been obtained from each patient before the surgery. The average age of the patients was 58.9 years, with a standard deviation of 12.87. Fifty percent of the studied sample fell between 56 and 67 years, with the youngest and oldest patients being 34 and 87 years, respectively. The sex distribution of the sample was 24 women (80%) and 6 men (20%) which coincides with the distribution of patients seen in the clinic. Of these cases, 18 (60%) affected the left foot and 12 (40%) the right.

The second metatarsal surgery was performed by the same surgeon in all cases. In the majority of patients, surgery of the second metatarsal, object of the present study, was accompanied by surgery of another metatarsal during the same surgical act, following the Leventen formula [24] and/or by proximal phalanx osteotomy and flexor tenotomy.

**Fig. 1:**
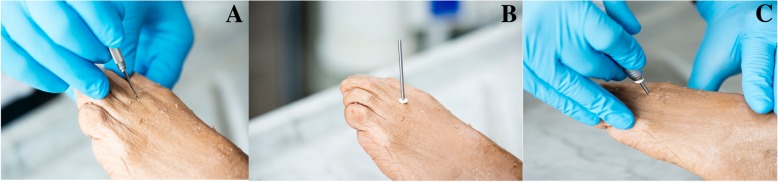
Surgical approach from dorsal-distal to plantar-proximal. Incision with Beaver (**a**); placement of the surgical Shannon with 45° angulation in the surgical neck of the second metatarsal (long Shannon) (**b**); and surgical gesture with which we perform the osteotomy that will allow the metatarsal to be shortened (**c**)

The incision was performed using Beaver 67 MIS surgical blades (Fig. 1a) and the raising and shortening osteotomy with a long straight Shannon (Fig. 1b, c). The Shannon was positioned at an angle of 45° relative to the long axis of the metatarsal, on the surgical neck of the affected bone, and the osteotomy was in a dorsal-distal to proximal-plantar direction. Once the surgery was completed, a post-surgical taping made of gauze bandage and Hypafix was applied as a measure of compression and fixation. The patient received a post-op shoe and gel bags for cryotherapy, according to the clinic protocol. After 72 hours, the patient went to the clinic for a fluoroscopy check and 10 days after surgery the post-surgical taping was changed for the first time. The new post-surgical taping consisted only of Hypafix and was changed weekly. Lastly, 4 weeks after surgery, both the post-surgical taping and the post-op shoe were removed. To evaluate the shortening of the metatarsal bone, measurements were made with electronic callipers from dorso-plantar and oblique X-ray plates taken both before and 6 months after the operation by the same radiologist.

The degree of functional recovery and the perception of well-being of the patient were studied by evaluating recorded pre- and postoperative means of the scale for minor metatarsals and interphalangeal joints of the American Orthopedic Foot and Ankle Society (AOFAS). All the patients had been given an AOFAS test to evaluate the improvement and the degree of satisfaction with the treatment received. In the cases in which several metatarsals were operated, the items of the scale in relation to pain, limitation in the activity, requirements in the footwear or alignment reflect the improvement after the set of interventions. However, for the items of the scale in relation to hyperkeratosis, dorsal-plantar flexion in the metatarsophalangeal joint or plantar flexion of the interphalangeal joint has been specifically asked in the survey for each of the metatarsals, and in the present work, the results belong to the tests that included in these items the data of the second metatarsal.

**Fig. 2:**
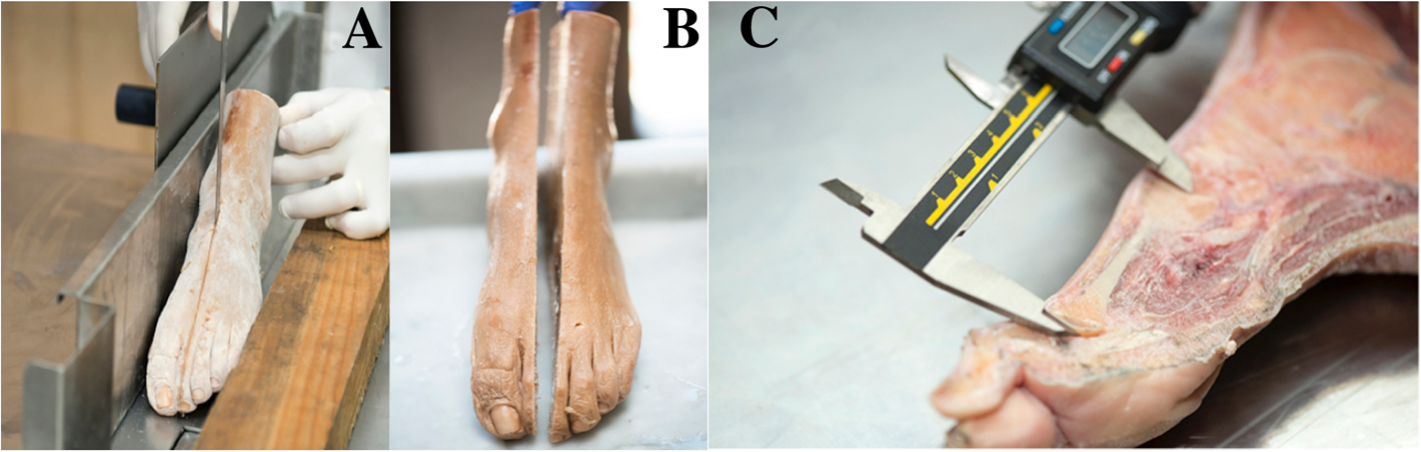
Cadaveric feet. Sagittal section of frozen foot following the diaphysis of the second metatarsal (**a** and **b**); exposure of structures and measurement by electronic caliber that assesses the shortening of the metatarsal to be treated (**c**)

The cadaveric feet (*n* = 10) were provided by the Anatomy Department of the Faculty of Medicine. Five were left feet and five were right. The surgical protocol was the same as that used with the patients. After surgery, the foot was frozen and then a sagittal section was made, passing through the second ray (Fig. 2a, b). The shortening of the metatarsal was then measured, again using electronic callipers (Fig. 2c). Finally, the integrity of the plantar plate was checked, meticulously examining each of its elements in detail.

For statistical analysis, the free software for statistical computing and graphic design R was used.

## Results

**Fig. 3:**
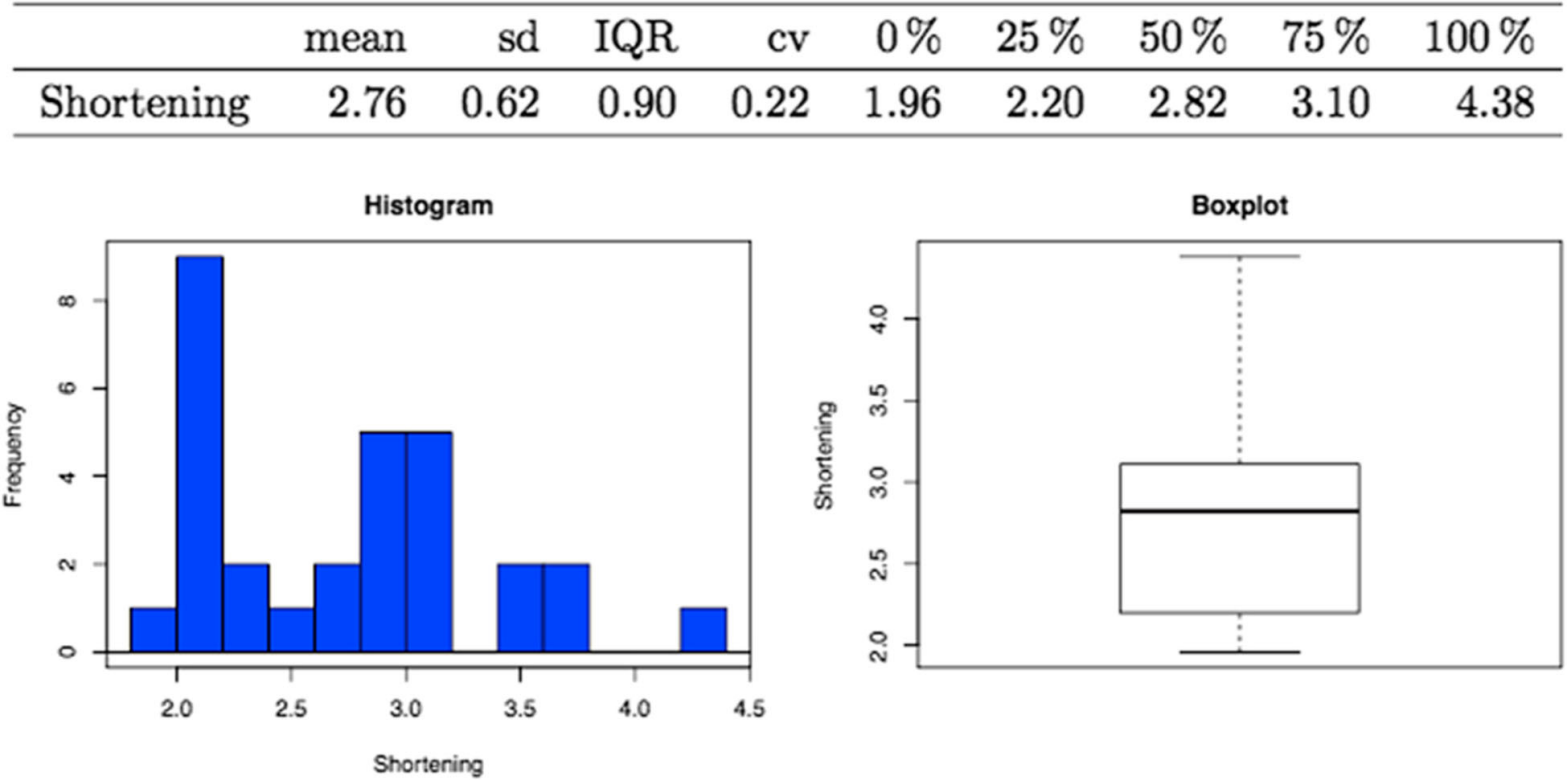
Mean shortening of the second metatarsal with graphic demonstration by histogram (left) and boxplot (right)

**Fig. 4:**
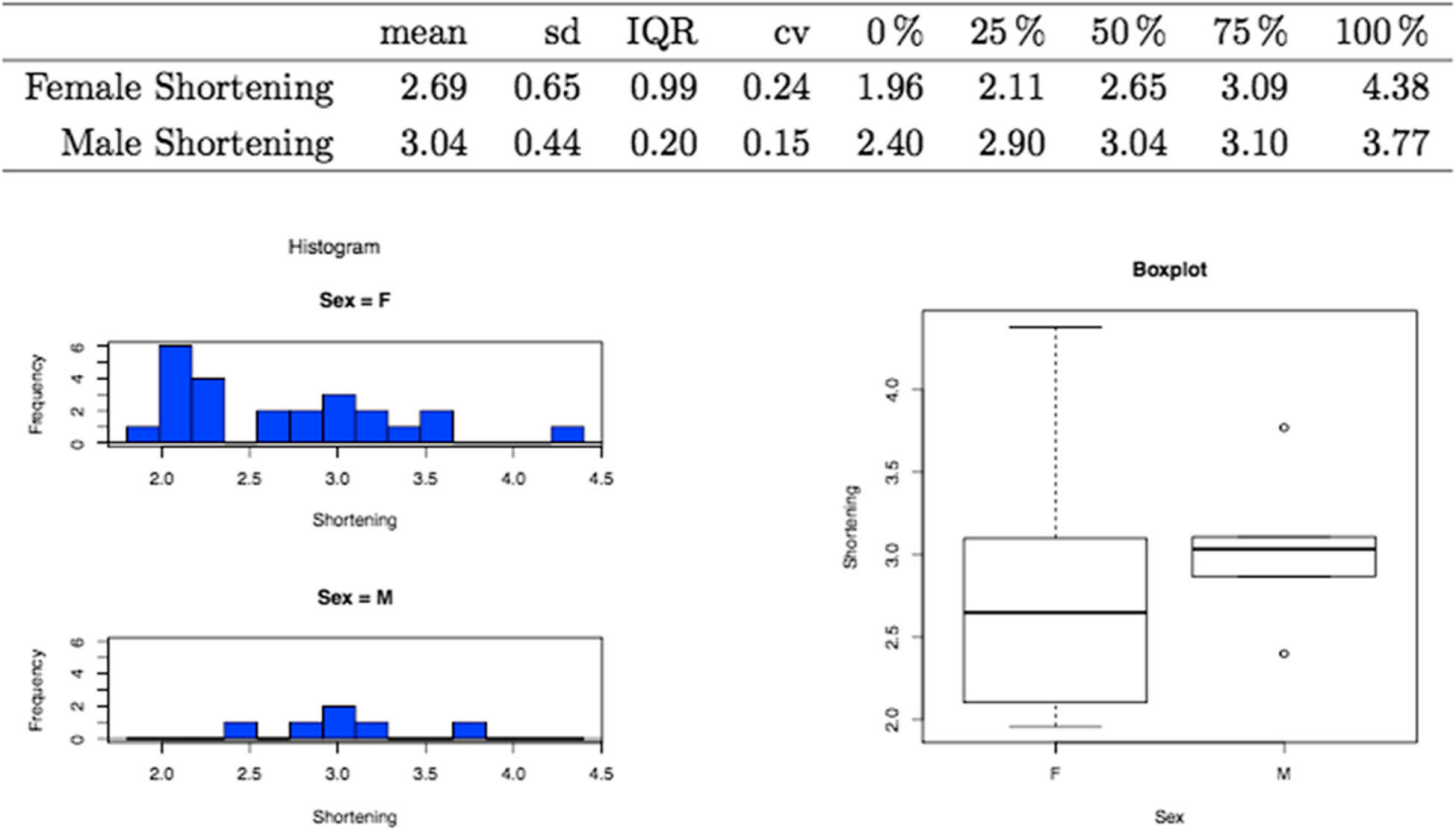
Mean shortening of the second metatarsal distributed by sexes with graphical demonstration by histogram (left) and boxplot (right)

**Fig. 5:**
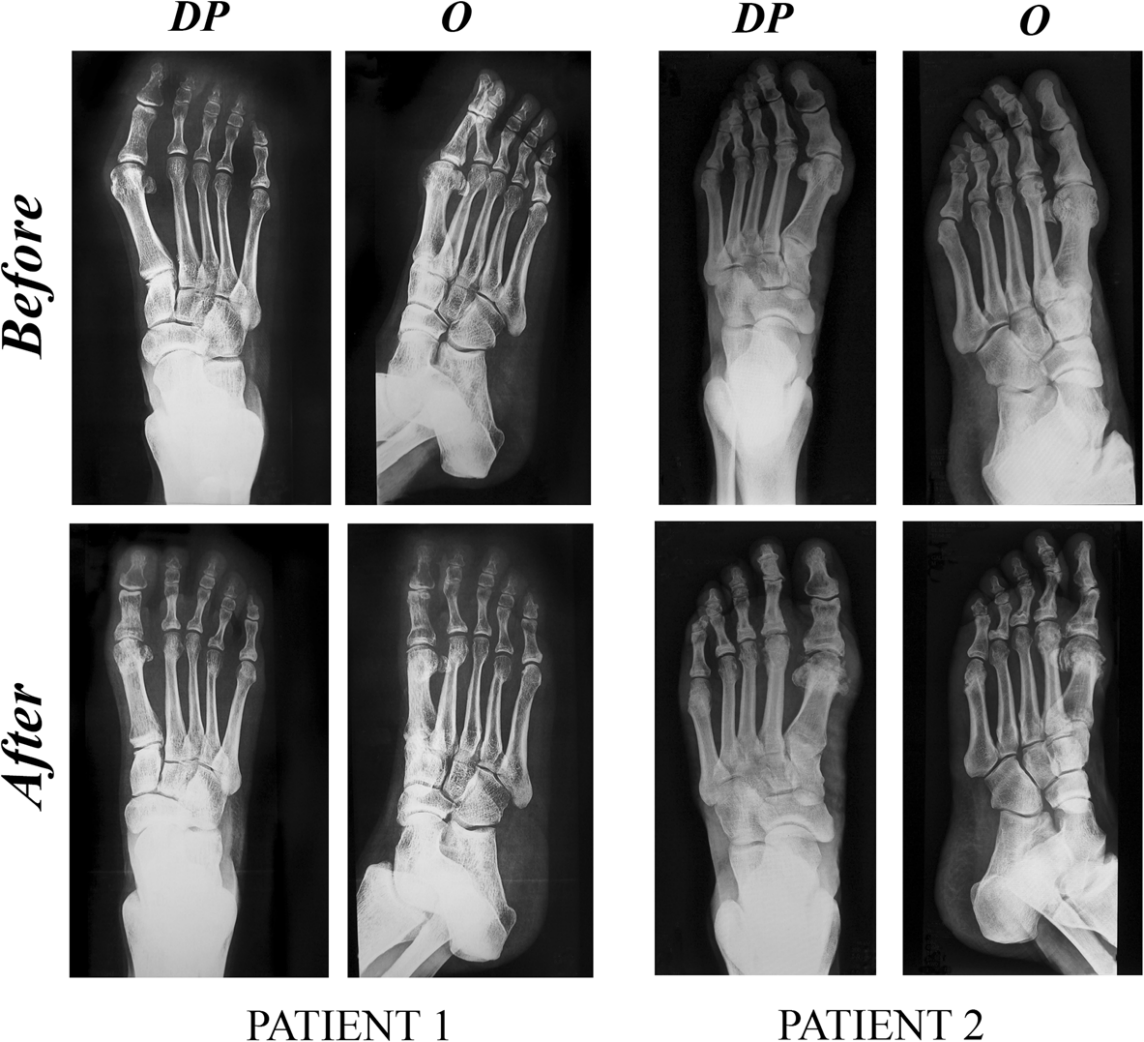
Examples of X-ray plates taken before and after surgical procedure from two patients (PATIENT 1 and PATIENT 2). DP dorsoplantar, O oblique

The mean reduction in length of the metatarsal in the cases included in this study (*n* = 30) was 2.76 ± 0.62 mm (Fig. 3). In the case of the male patients, the mean was higher (3.04 ± 0.2 mm) than in the females (2.69 ± 0.99 mm) (Fig. 4). Examples of X-ray plates taken before and after the surgical procedure from two patients are showed in Fig. 5.

The average index of satisfaction as measured by the AOFAS metatarsal scale was 50.3 before and 95.26 after surgery, with an average follow-up period of 1.5 years. Of note is the lack of stiffness or alterations in the stability of the MTP joint, as well as the lack of transfer metatarsalgia. No patient showed malunion, delayed union or no union.

**Fig. 6:**
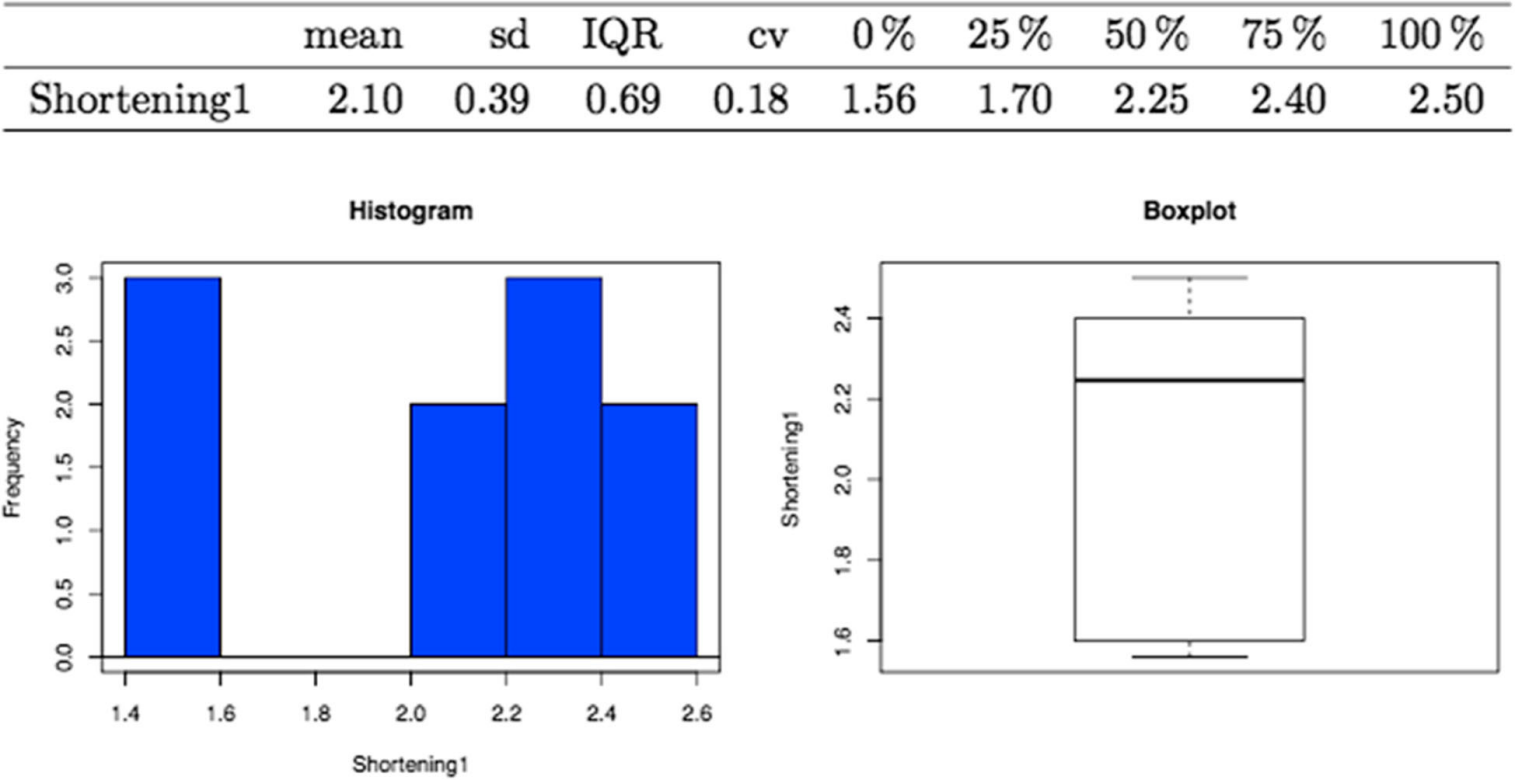
Mean shortening of the second metatarsal in cadaveric feet with graphical demonstration by histogram (left) and boxplot (right)

**Fig. 7:**
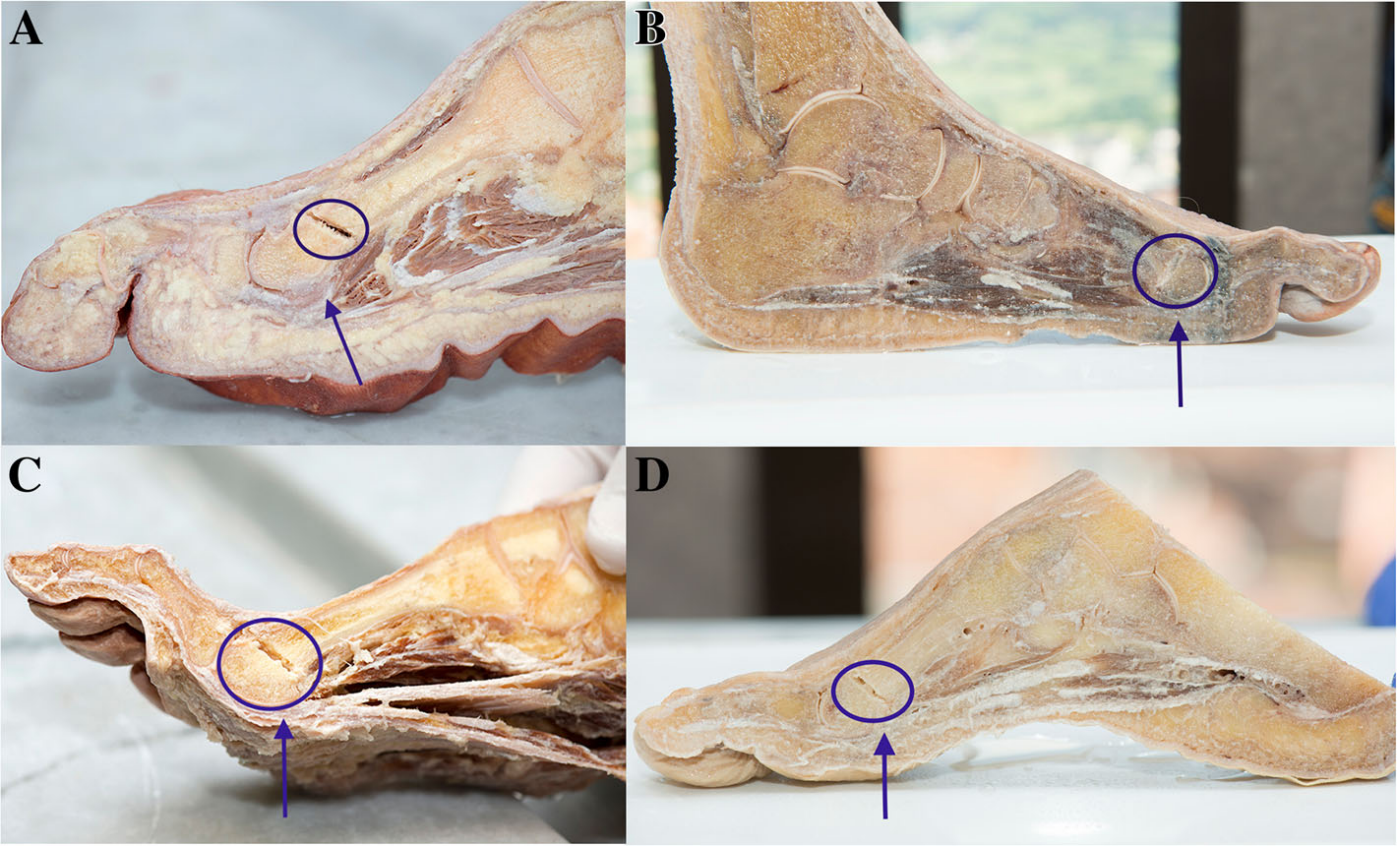
Examples of cadaveric feet (**a**-**d**) where the osteotomy performed in the second metatarsal (circle) and the preservation of the plantar plate after surgery (arrow) can be observed

In the case of the cadaveric feet (*n* = 10), the mean shortening due to surgery was 2.10 ± 0.39 mm, the highest value being 2.50 mm (Fig. 6), and in all of these feet, the integrity of the plantar plate and soft tissues was seen to be perfectly intact (Fig. 7).

## Discussion

One of the results of the present study that provides most reassurance about the use of MIS surgery for the treatment of metatarsalgia is the high score achieved on the AOFAS scale, since, as previously mentioned, there are many alternative therapies for the treatment of metatarsalgias of the central rays. Among the options described in the medical literature are reports of good results obtained by open surgical techniques, such as the Weil osteotomy.

Using this procedure, Ruiz Ibán [25] obtained good or excellent results in 87.5% of cases, operating on 40 subjects, with a mean follow-up of 2.3 years, and achieving an average AOFAS scale score of 85 points. Hofstaetter [26] obtained good results in 88% of cases, based on a group of 24 subjects with a mean follow-up of 7 years, and an average AOFAS score of 83 points. Also, Henry et al [27] achieved an average AOFAS score of 85.3. Major frequent complications in these studies refer to transfer metatarsalgia and residual metatarsalgia.

In our study, using distal metatarsal osteotomy performed by means of MIS surgery, the average score on the AOFAS scale was 95.26 points (75 the lower and 100 the higher scores). Although this scale is not validated and so may not totally reflect the real functional results [28], many clinicians continue to administer the AOFAS survey to patients and it is widely used in the literature, offering a good comparison between different studies [29]. Our results, according to Botezatu et al. [14], strongly support distal metatarsal osteotomy by MIS for the treatment of metatarsalgia of the lesser rays. They show that it gives good clinical and aesthetic results, without the appearance of stability problems or rigidity of the MTP joint, transfer metatarsalgia or other complications. The good consolidation and lack of malunions, delayed unions or no unions also support the indication of this type of surgery. The complications of the Weil osteotomy, on the other hand, are relatively high, and a literature review recently undertaken includes the presence of floating toes, recurrence, transfer metatarsalgia and a few delayed unions and no unions [30]. Although there are few comparative studies between the two types of surgery, a recent publication indicates the greater range of motion (ROM) in the metatarsophalangeal joint after the osteotomy by MIS than after the Weil osteotomy [31], whereas Jarde et al. [32] reported a series of Weil’s osteotomies where they found reduced mobility of the MP joint in all cases.

In the majority of cases, this metatarsalgia is accompanied by hyperkeratosis of the most affected area. As the principal objective of metatarsalgia treatment is the elimination of pain, the existence of persistent asymptomatic hyperkeratosis cannot be considered a failure of treatment, so long as it is painless. Migues [33] and Hofstaetter [26] described painful hyperkeratosis in 16% and 12%, respectively, of Weil osteotomy patients. In the present study, painful hyperkeratosis did not persist in any of the cases, although three patients suffered asymptomatic hyperkeratosis and were included in the AOFAS scale as failures. We believe that, in agreement with De Prado et al. [34], osteotomies should be performed according to the criteria of the Leventen formula [24]. That is to say, osteotomies of the second and third metatarsals should be performed when the second metatarsal is affected; osteotomies of the second to fourth metatarsals should be carried out in the case of metatarsalgias in the area of the third metatarsal; and osteotomies of the third to fourth metatarsals in the case of metatarsalgias in the area of the fourth metatarsal.

The average shortening of the metatarsal in the present study (2.76 mm) is less than that obtained by Weil’s open surgery [20, 25–27, 33]. Shortening results are similar to other recently published studies employing MIS [35]. The metatarsal parabola proposed by Maestro [36] is not always achieved. However, the objective of the osteotomy in the treatment of metatarsalgia is the shortening of the metatarsal together with the raise of its head in order to off-load the forefoot. After percutaneous surgery, there is, additionally, a re-positioning of the metatarsal heads as a result of tension of soft tissues. Finally, on full weight-bearing, the metatarsal heads gain the best position for weight distribution [37]. All these factors produce an off-load of the forefoot with decreased pressure on the metatarsal heads that, ultimately, was causing the pain.

Despite the fact that in the present series, the degree of shortening seen in the male patients (3.4 mm) is greater than in the females (2.69 mm), the greatest reduction in length (4.38 mm) was produced in a female patient. When the shortening of the affected metatarsal is greater than 4 mm, there are quite a few complications, such as stiffness or floating toes [38]. However, this patient was the oldest in the study, which suggests that a variety of factors may influence the degree of shortening produced by the osteotomy, one of which could be the presence of osteoporosis.

Careful observation of the plantar plate and soft tissues around the metatarsal head in cadaver feet indicates that there is no damage to these tissues during surgery. Although these observations were made in cadaveric feet, where the scenario is different from the clinical, we may consider this as a simple approach for the study of these tissues. The fact that it was the same surgeon who performed the operation guarantees that it has been carried out in the same way as it was done on the feet of the patients.

## Conclusions

The fundamental conclusion drawn from this study is that after MIS surgery, the metatarsal shortening is less than that achieved in open surgery; however, this smaller shortening is compensated likely by the metatarsal head elevation, the tension of unharmed soft tissues and the full weight-bearing, finally achieving an excellent patient satisfaction index regarding AOFAS results (with the caution that should be taken when using no patient-reported outcome (PRO) measures). The absence of damage to the plantar plate and surrounding soft tissues is another important outcome (always considering that the experimental model is a different scenario as the clinical one), since it has not been possible to find any reference to the preservation of its structural integrity in previous publications describing studies whatever surgical protocol used. As a result, this can be described as an effective, safe and recommendable technique, whether or not it is associated with other open or MIS surgical forefoot procedures.

## Abbreviations

AOFAS: American Orthopedic Foot and Ankle Society
MIS: Minimal invasive surgery
MTP: Metatarsophalangeal
